# Surgical treatment of scoliosis: a review of techniques currently applied

**DOI:** 10.1186/1748-7161-3-6

**Published:** 2008-04-18

**Authors:** Toru Maruyama, Katsushi Takeshita

**Affiliations:** 1 Department of Orthopaedic Surgery, Saitama Medical Center, Saitama Medical University, Saitama, Japan; 2 Department of Orthopaedic Surgery, Faculty of Medicine, The University of Tokyo, Tokyo, Japan

## Abstract

In this review, basic knowledge and recent innovation of surgical treatment for scoliosis will be described. Surgical treatment for scoliosis is indicated, in general, for the curve exceeding 45 or 50 degrees by the Cobb's method on the ground that:

1) Curves larger than 50 degrees progress even after skeletal maturity.

2) Curves of greater magnitude cause loss of pulmonary function, and much larger curves cause respiratory failure.

3) Larger the curve progress, more difficult to treat with surgery.

Posterior fusion with instrumentation has been a standard of the surgical treatment for scoliosis. In modern instrumentation systems, more anchors are used to connect the rod and the spine, resulting in better correction and less frequent implant failures. Segmental pedicle screw constructs or hybrid constructs using pedicle screws, hooks, and wires are the trend of today.

Anterior instrumentation surgery had been a choice of treatment for the thoracolumbar and lumbar scoliosis because better correction can be obtained with shorter fusion levels. Recently, superiority of anterior surgery for the thoracolumbar and lumbar scoliosis has been lost. Initial enthusiasm for anterior instrumentation for the thoracic curve using video assisted thoracoscopic surgery technique has faded out.

Various attempts are being made with use of fusionless surgery. To control growth, epiphysiodesis on the convex side of the deformity with or without instrumentation is a technique to provide gradual progressive correction and to arrest the deterioration of the curves. To avoid fusion for skeletally immature children with spinal cord injury or myelodysplasia, vertebral wedge ostetomies are performed for the treatment of progressive paralytic scoliosis. For right thoracic curve with idiopathic scoliosis, multiple vertebral wedge osteotomies without fusion are performed. To provide correction and maintain it during the growing years while allowing spinal growth for early onset scoliosis, technique of instrumentation without fusion or with limited fusion using dual rod instrumentation has been developed. To increase the volume of the thorax in thoracic insufficiency syndrome associated with fused ribs and congenital scoliosis, vertical expandable prosthetic titanium ribs has been developed.

## Review

Considering that not all the scoliosis patients can be treated successfully with conservative treatment and severe and/or progressive scoliosis often need surgery, even the specialists of conservative treatment should have knowledge about surgical treatment. In this review, basic knowledge and recent innovation of surgical treatment for scoliosis will be described. Because relatively little data are obtained regarding outcomes in the long-term or clinical outcomes such as patients' satisfaction, the particular techniques will be discussed mainly based on the radiological outcomes in the middle-term, sometimes short-term follow-up.

### Indication of surgery

Surgical treatment for scoliosis is indicated, in general, for the curve exceeding 45 or 50 degrees by the Cobb's method on the ground that:

1) Curves larger than 50 degrees progress even after skeletal maturity. Thoracic curves with magnitude between 50 and 75 degrees at skeletal maturity (Risser IV or V) progressed of an average of 29.4 degrees over the 40.5 years follow-up period [1]. Curves larger than 55 degrees at skeletal maturity (partial or total fusion of the completed iliac apophyses) progressed of more than 0.5 degrees per year [2]. Thoracic curves with an average Cobb angle of 60.5 dgrees progressed to 84.5 degrees over the 50 years follow-up period [3].

2) Curves of greater magnitude cause loss of pulmonary function, and much larger curves cause respiratory failure. In patients with curves between 60 and 100 degrees, total lung capacity was 68% of predicted normal values [4]. Nearly half of the patients with thoracic curve larger than 80° degrees had shortness of breath at the average age of 42 years [5]. Vital capacity below 45% predicted and a Cobb angle greater than 110 degrees were risk factors to develop respiratory failure and earlier death [6].

3) Larger the curve progress, more difficult to treat with surgery: more surgical anchors may be necessary, longer operation time, more blood loss, higher surgical complication rate may be expected.

Sometimes patient's motivation to straighten her/his spine by surgery should be respected, especially for the patient with gray zone curve, Cobb angle of 40 to 45 degrees.

Surgical treatment for scoliosis can be divided into fusion surgery and fusionless surgery.

### Fusion surgery

#### Posterior instrumentation

**Figure 1:**
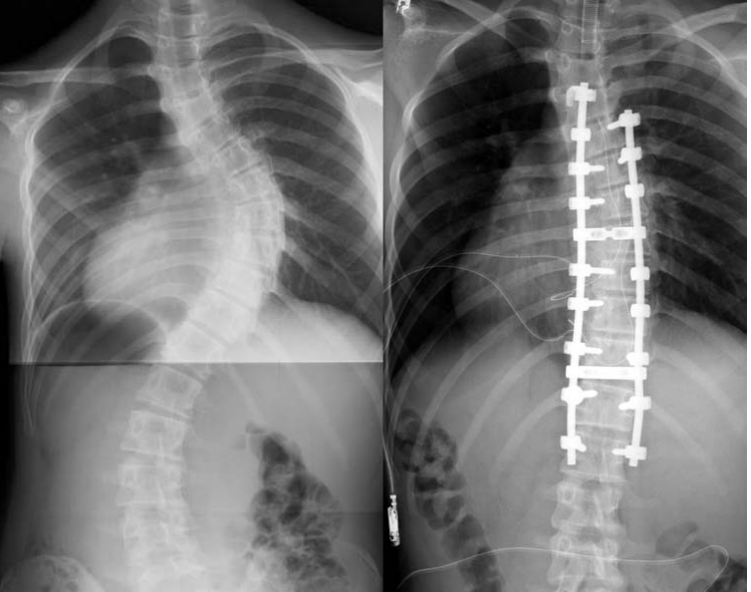
**Segmental pedicle screw constructs**. Right thoracic curve between the T5 and T11 was corrected from 68 to 25 degrees.

**Figure 2:**
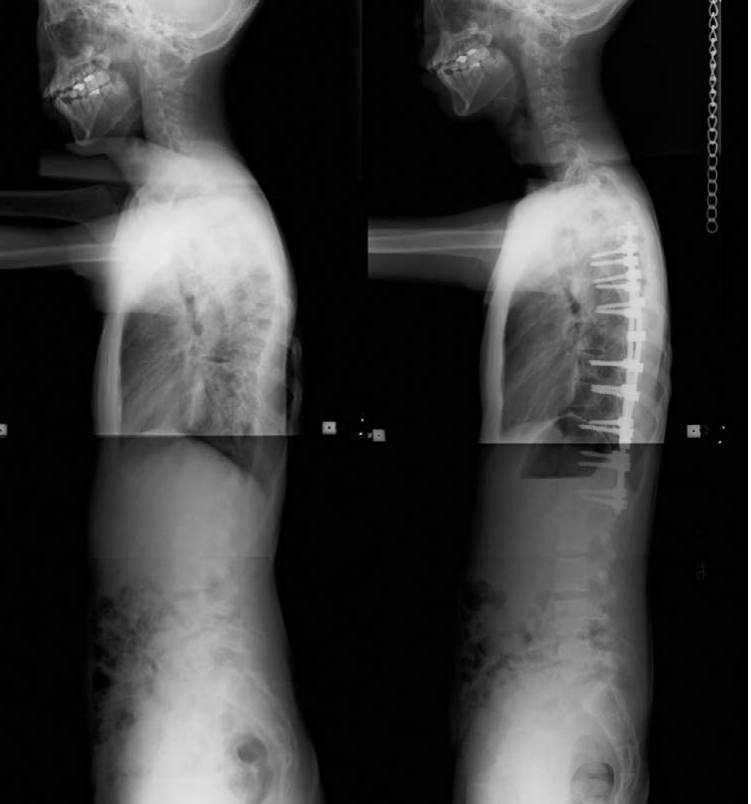
**Segmental pedicle screw constructs**. Lateral radiographs before and after surgery.

**Figure 3:**
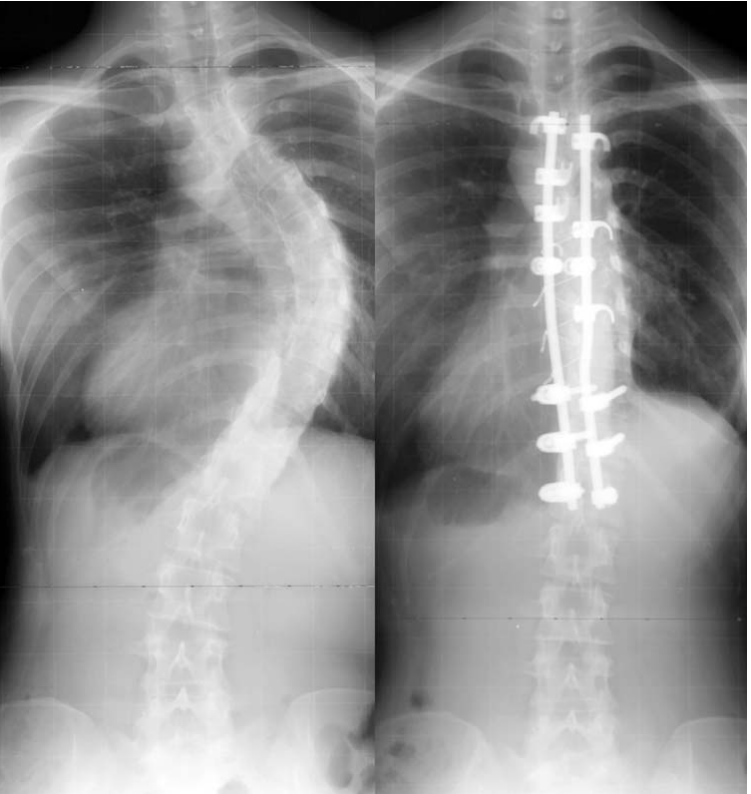
**Hybrid constructs using pedicle screws, hooks, and wires**. Right thoracic curve between the T5 and T11 was corrected from 70 to 23 degrees.

**Figure 4:**
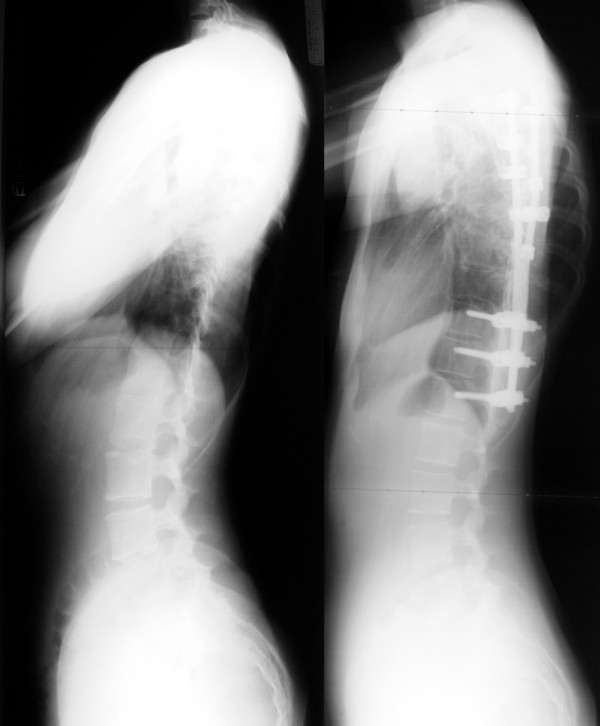
**Hybrid constructs using pedicle screws, hooks, and wires**. Lateral radiographs before and after surgery.

Posterior fusion with instrumentation has been a standard of the surgical treatment for scoliosis since first introduced by Paul Harrington [7]. In his system, correction force was applied with distraction along the concavity of the curve. In the second generation instrumentation system developed by Cotrel and Dubousset [8], correction was attempted by the rod-rotation maneuver. In modern instrumentation systems, more anchors are used to connect the rod and the spine, resulting in better correction and less frequent implant failures [9]. Segmental pedicle screw constructs (Fig. 1, 2) or hybrid constructs using pedicle screws, hooks, and wires (Fig. 3, 4) are the trend of today.

A segmental pedicle screw concept was first introduced by Suk [10]. He reported that the idiopathic thoracic curves of 51 degrees in average were corrected to 16 degrees (69% correction) with a minimum 5-year follow-up. Although 1.5% of the screws inserted in the thoracic level were malpositioned, they did not cause neurologic complications or adversely affect the long-term results. Asher et al. [11] reported on 63% correction with a minimum 5-year follow-up using hybrid constructs with hooks, apical sublaminar wires, and pedicle screws. In 2005, Cheng et al. [12] compared apical sublaminar wires with pedicle screws. No difference was found regarding initial correction (67.4% vs. 68.1%), loss of correction (4.6% vs. 5.1%), operating time (350 minutes vs. 357 minutes), satisfaction of the patients, but intraoperative blood loss was more with wires (1791 ml vs. 824 ml) and instrumentation cost was higher with screws (8341 USD vs. 13462 USD). Another concern with segmental pedicle screw constructs is that vigorous correction of a major curve is an overcorrection relative to the flexibility of the upper compensatory curve [13]. Generally, an extent of fusion level is determined with the flexibility of the curves demonstrated on the radiographs taken in supine side bending, fulcrum side bending, traction, or push-prone position [14-16]. With segmental pedicle screw technique, to avoid the postoperative shoulder imbalance, frequently fusion has to be extended to the upper thoracic vertebrae, which is not included in the fusion with other techniques.

#### Anterior instrumentation

**Figure 5:**
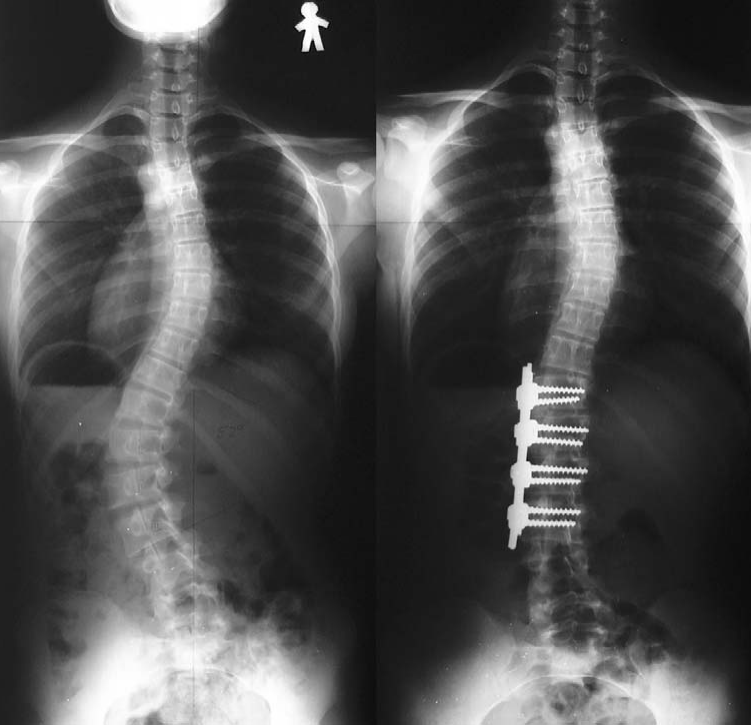
**Anterior instrumentation surgery**. Left thoracolumbar curve between the T11 and L4 was corrected from 52 to 19 degrees (By courtesy of Dr. Tomasz Kotwicki).

**Figure 6:**
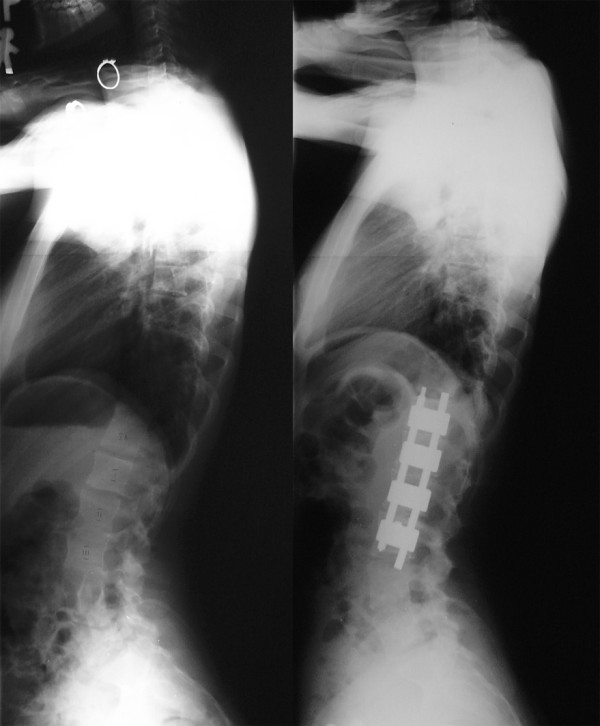
**Anterior instrumentation surgery**. Lateral radiographs before and after surgery (By courtesy of Dr. Tomasz Kotwicki).

Anterior instrumentation surgery (Figure 5, 6) had been a choice of treatment for the thoracolumbar and lumbar scoliosis because better correction can be obtained with shorter fusion levels. Moreover, anterior instrumentation for the thoracic curve using video assisted thoracoscopic surgery technique had been developed [17]. Initial enthusiasm for this surgery in expectation of decreased postoperative pain or patients' satisfaction with less operative scar has faded out because the thoracic aorta is at risk if screw penetrated the cortex on the opposite side [18,19], and disruption of the chest cage during the surgical treatment affects the pulmonary function after surgery [20]. Thoracic curve can be treated successfully with posterior instrumentation surgery without affecting pulmonary function. In 2005, Potter et al. [21] compared anterior spinal fusion and posterior spinal fusion for the treatment of single thoracic curve, and concluded that posterior fusion group demonstrated greater curve correction (62% versus 52%) and greater rib hump correction (51% versus 26%). Recently, superiority of anterior surgery for the thoracolumbar and lumbar scoliosis has been lost. In 2007, Hee et al. [22] compared segmental pedicle screw instrumentation and anterior instrumentation in adolescent idiopathic thoracolumbar and lumbar scoliosis. They reported that the coronal correction at a minimum 2-year follow-up was compatible (68% vs. 67%), but length of surgery was significantly shorter (189 minutes vs. 272 minutes) and length of hospital stay was shorter (6.2 days vs. 8 days) in the posterior segmental pedicle screw group.

### Fusionless surgery

Various attempts are being made with use of fusionless surgery to control growth, to avoid fusion, to delay the timing of the definitive fusion surgery, or to increase the volume of the thorax.

#### To control growth

Epiphysiodesis on the convex side of the deformity with or without instrumentation is a technique to provide gradual progressive correction and to arrest the deterioration of the curves. Marks et al. [23] found anterior and posterior growth arrest alone not effective to prevent progression of deformity in infantile scoliosis. To the contrary, Betz et al. [24] showed that stapling the anterior vertebral spinal growth plates could control growth of the curve with adolescent idiopathic scoliosis. By using newly designed biocompatible shape memory metal alloy staples, 6 of 10 patients with average curve magnitude of 35 degrees were stabilized during more than 1-year follow-up period. To avoid the overtreatment for relatively small, non-progressive curve with this technique, definite and solid criteria for hallmarking a curve as progressive should be established first.

#### To avoid fusion

By fusion surgery, segmental motion of the vertebral column is eliminated. To avoid fusion for patients with paralysis, for whom maintaining spinal flexibility and mobility is more desirable, fusionless, vertebral wedge ostetomies are developed for the treatment of progressive paralytic scoliosis of skeletally immature children with spinal cord injury or myelodysplasia [25]. A specially designed implant system is used to assist with correction and maintenance of alignment. Twelve weeks following the initial surgery, a second surgery is necessary to remove parts of the implants. This technique may be used for idiopathic scoliosis in future.

**Figure 7:**
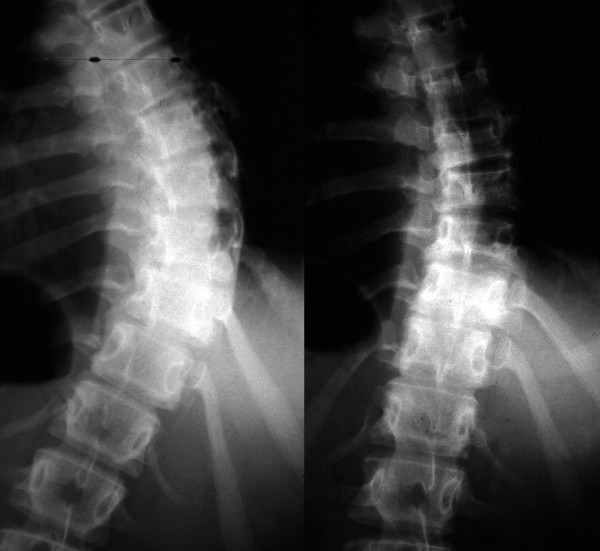
**Multiple vertebral wedge osteotomy**. Right thoracic curve between the T5 and T12 corrected from 56 to 26 degrees.

**Figure 8:**
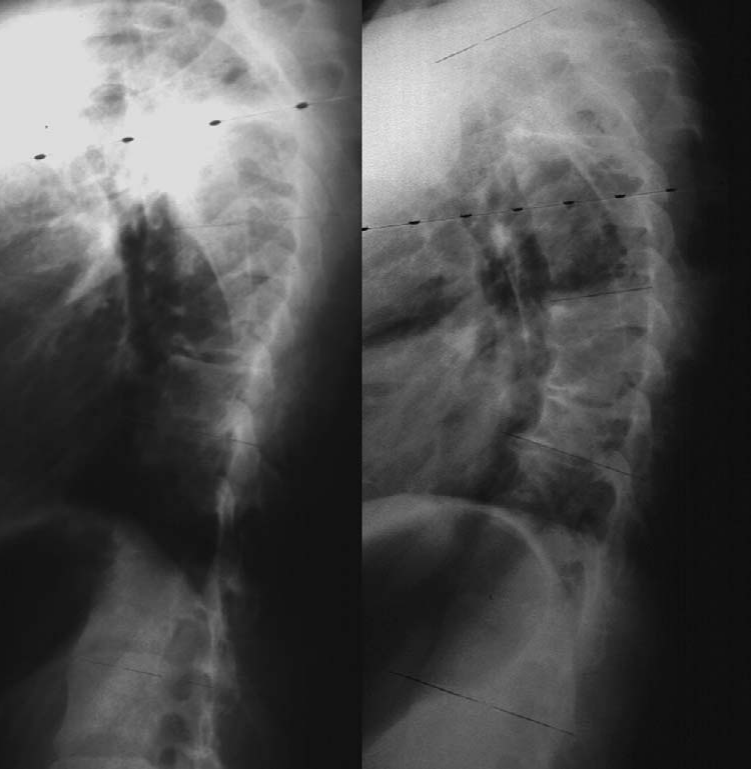
**Multiple vertebral wedge osteotomy**. Lateral radiographs before and after surgery.

For right thoracic curve with idiopathic scoliosis, multiple vertebral wedge osteotomies without fusion (Fig. 7, 8) are performed [26]. Twenty patients were treated with osteotomies on the averaged 3.6 periapical vertebrae and followed-up for 8.9 years on an average. There were no neurologic complications. For four patients with Risser 0 or I, average curve magnitude was 74.8 degrees before surgery and 67.5 degrees at the latest follow-up (correction rate was 9.8%), whereas, for 16 patients with Risser IV or V, that was 61.3 degrees before surgery and 43.3 degrees at the latest follow-up (correction rate was 29.4%).

#### To delay the timing of fusion

Fusion surgery in very young age results in the short trunk relative to the extremities. It also affects the development of the lung. To provide correction and maintain it during the growing years while allowing spinal growth for early onset scoliosis, technique of instrumentation without fusion or with limited fusion using Harrington rod, Cotrel-Dubousset rod, or Luque rod had been developed [27,28]. Recently, Akbarnia et al. [29] developed the technique using Isola dual rod instrumentation. Upper and lower foundations are made bilaterally using hooks or pedicle screws as anchoring devices. Each foundation is connected to a rod, and the rods are connected by a tandem connector, which is placed at the thoracolumbar junction on each side. Lengthening is performed usually every 6 months by distraction inside the tandem connector or between the rod and the tandem connector. Once maximum spinal growth is accomplished, definitive final arthrodesis with instrumentation is performed. Between 1993 and 2001, 23 patients with various etiologies underwent this treatment at an average age of 5.4 years. The averaged curve magnitude was 82 degrees before surgery, 38 degrees after the initial surgery, and 36 degrees after 6.6 times of lengthening procedures. The length of thoracic and lumbar spine increased by 5 cm at the initial surgery and 4.7 cm in addition during the lengthening period.

#### To increase the volume of the thorax

To treat thoracic insufficiency syndrome associated with fused ribs and congenital scoliosis, vertical expandable prosthetic titanium ribs (VEPTR) has been developed [30]. After opening-wedge thoracostomy, the acute correction is stabilized by the device. The device is extended from the cephalad rib to the caudal rib, to the lumbar spine, or to the posterior iliac crest. Following the initial implantation, the devices are expanded at scheduled intervals of four to six months. Twenty-seven patients underwent surgery at the average age of 3.2 years and were followed-up for 5.7 years. Vital capacity significantly increased; moreover, scoliosis deformity was indirectly corrected from 74 to 49 degrees at the last follow-up.

## Conclusion

We described the indication of surgical treatment for scoliosis, results of the innovative surgical techniques, in terms of, posterior fusion with instrumentation, anterior fusion with instrumentation, and various kinds of fusionless surgery.

## Competing interests

The authors declare that they have no competing interests.

## Authors' contributions

TM conceived of the study, participated in its design and drafted the manuscript. KT participated in the design of the study and helped to draft the manuscript. All authors read and approved the final manuscript.
